# Melanic pigmentation and light preference within and between two *Drosophila* species

**DOI:** 10.1002/ece3.7998

**Published:** 2021-08-12

**Authors:** Arielle M. Cooley, Suzanne Schmitz, Eduardo J. Cabrera, Mitchell Cutter, Maxwell Sheffield, Ian Gingerich, Gabriella Thomas, Calvin N. M. Lincoln, Virginia H. Moore, Alexandra E. Moore, Sarah A. Davidson, Nikhil Lonberg, Eli B. Fournier, Sophia M. Love, Galen Posch, Matthew B. Bihrle, Spencer D. Mayer, Kuenzang Om, Lauren Wilson, Casey Q. Doe, Chantalle E. Vincent, Elizabeth R. T. Wong, Ilona Wall, Jarred Wicks, Stephon Roberts

**Affiliations:** ^1^ Biology Department Whitman College Walla Walla WA USA

**Keywords:** behavioral choice experiment, correlated traits, *Drosophila americana*, *Drosophila novamexicana*, *ebony*, histamine, light preference, melanin, pigmentation, pleiotropy, *tan*, vision

## Abstract

Environmental adaptation and species divergence often involve suites of co‐evolving traits. Pigmentation in insects presents a variable, adaptive, and well‐characterized class of phenotypes for which correlations with multiple other traits have been demonstrated. In *Drosophila*, the pigmentation genes *ebony* and *tan* have pleiotropic effects on flies' response to light, creating the potential for correlated evolution of pigmentation and vision. Here, we investigate differences in light preference within and between two sister species, *Drosophila americana* and *D. novamexicana*, which differ in pigmentation in part because of evolution at *ebony* and *tan* and occupy environments that differ in many variables including solar radiation. We hypothesized that lighter pigmentation would be correlated with a greater preference for environmental light and tested this hypothesis using a habitat choice experiment. In a first set of experiments, using males of *D. novamexicana* line N14 and *D. americana* line A00, the light‐bodied *D. novamexicana* was found slightly but significantly more often than *D. americana* in the light habitat. A second experiment, which included additional lines and females as well as males, failed to find any significant difference between *D. novamexicana*‐N14 and *D. americana*‐A00. Additionally, the other dark line of *D. americana* (A04) was found in the light habitat more often than the light‐bodied *D. novamexicana*‐N14, in contrast to our predictions. However, the lightest line of *D. americana*, A01, was found substantially and significantly more often in the light habitat than the two darker lines of *D. americana*, thus providing partial support for our hypothesis. Finally, across all four lines, females were found more often in the light habitat than their more darkly pigmented male counterparts. Additional replication is needed to corroborate these findings and evaluate conflicting results, with the consistent effect of sex within and between species providing an especially intriguing avenue for further research.

## Graphical abstract

Light preference was examined in the light‐bodied *Drosophila novamexicana* and in three lines of the dark‐bodied *Drosophila americana*. In both species, female flies are more lightly pigmented than male flies and also show a stronger tendency to select a more brightly lit habitat. Patterns of habitat choice within and between species were mixed, but showed some trends of lighter bodied flies preferring more brightly lit habitats.

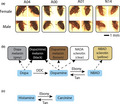

## 1 INTRODUCTION

Correlations among phenotypic traits are ubiquitous, with profound implications for the evolution of populations (Lande and Arnold, 1983). Although phenotypic correlations are frequently observed in nature, the underlying causes are potentially numerous and are often unknown (Endler, 1986; Stearns, 1992). Traits can be genetically correlated due to either linkage or pleiotropy, while genetically unassociated traits may evolve in a correlated fashion due to “selective covariance,” in which selection tends to act simultaneously on two or more traits (Armbruster & Schwaegerle, 1996). Finally, populations and species can diverge from one another in suites of traits due simply to the unique history of mutation, migration, and drift within each group (Armbruster & Schwaegerle, 1996).

One trait that frequently evolves as part of a suite of correlated characters is pigmentation. In the model insect genus *Drosophila*, correlations due to pleiotropy of an underlying gene have been reported for pigmentation and trichome patterns (Gompel & Carroll, 2003) and for pigmentation and vision (True et al., 2005). Selective covariance is also likely to influence patterns of pigment evolution in *Drosophila*: altitudinal and latitudinal gradients in melanic pigmentation have been documented in multiple species and have been ascribed to selection associated with heat, ultraviolet radiation, and/or humidity (Clusella Trullas et al., 2007; Pool & Aquadro, 2007; Rajpurohit & Nedved, 2013; Rajpurohit et al., 2008; Telonis‐Scott et al., 2011; True, 2003). Thus, pigmentation in *Drosophila* is a promising system for investigating both genetic and environmental influences on the evolution of correlated traits.

**FIGURE 1:**
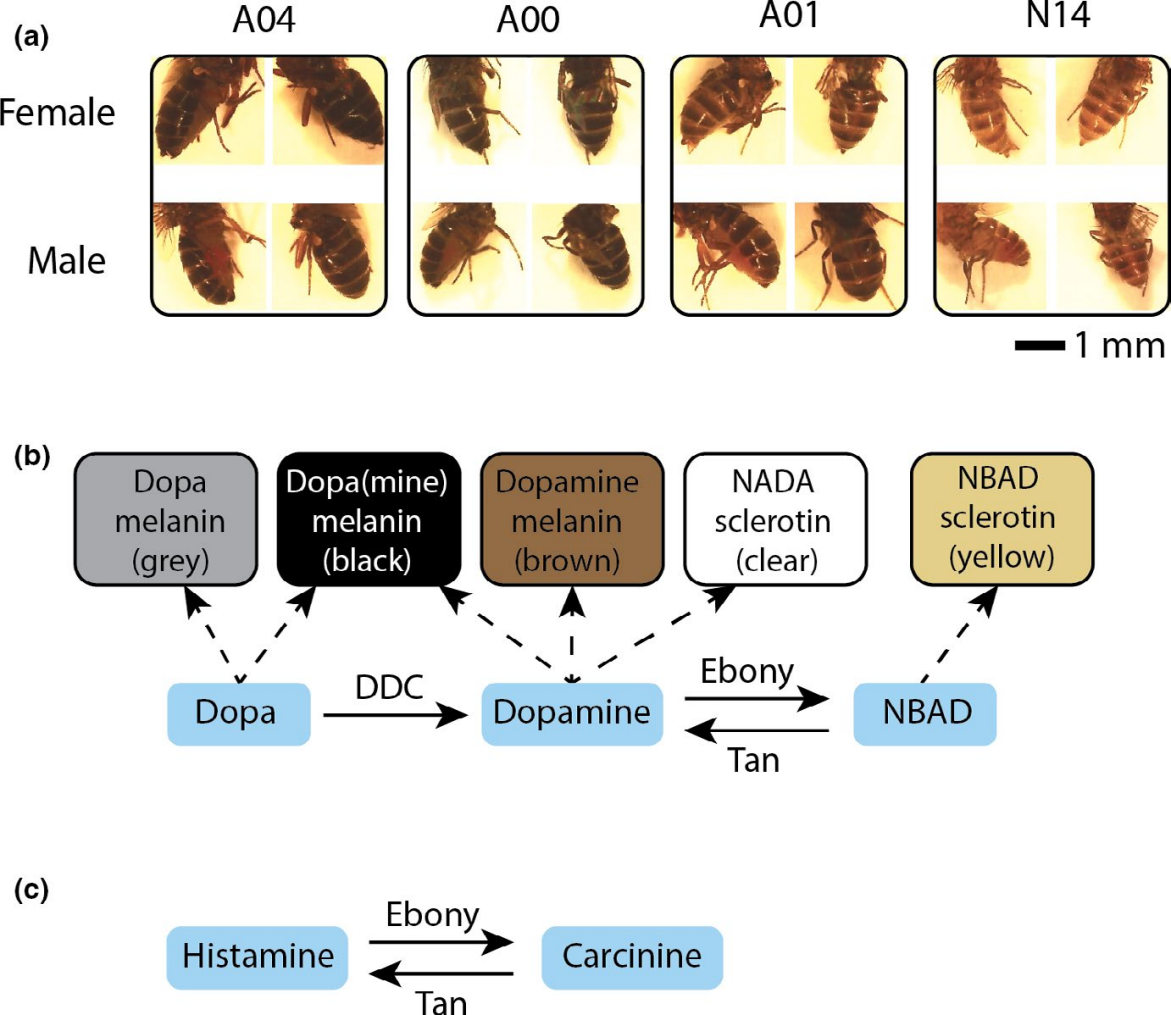
*Drosophila americana* and *D. novamexicana* differ in abdominal pigmentation, a trait influenced by the pleiotropic genes *ebony* and *tan*. (a) Female and male flies of *D. americana* (lines A04, A00, and A01) and *D. novamexicana* (line N14). Young adult flies of each taxon were collected and photographed in 2021, within a single 2‐hr period under constant lighting conditions. In each case, the lateral view (left) and the dorsal view (right) show the same individual. (b) The balance of *ebony* and *tan* expression helps determine cuticular pigmentation. (c) The same genes, *ebony* and *tan*, also participate in histamine recycling in the visual system. (b) and (c) are redrawn from Takahashi (2013)

While most of the documented pigmentation clines in *Drosophila* are altitudinal or latitudinal, a unique longitudinal gradient has been observed in *Drosophila americana*, with very dark brown flies found in the eastern United States and lighter flies found as far west as the Rocky Mountains (Throckmorton, 1982). Sister species *D. novamexicana* features an evolutionarily derived, lighter, and yellower body color, and its geographical distribution in the desert Southwest of the United States makes it appear to be a geographic extension of the pigmentation cline in *D. americana* (Wittkopp et al., 2009). Pigmentation in *D. novamexicana* is also highly variable, but it is always lighter than even the lightest lines of *D. americana* (Davis & Moyle, 2019). In addition to these patterns of variation within and between species (Figure 1a), female *D. americana* have been shown to be slightly lighter in color compared to males of the same lines despite a lack of difference in color patterning (Wittkopp et al., 2011).

The *D. americana*–*D. novamexicana* species pair, part of the dark‐bodied *virilis* group of *Drosophila*, diverged approximately 0.4 MYA (Caletka & McAllister, 2004; Morales‐Hojas et al., 2011). Two QTLs together explain 87% of the pigmentation difference between *D. americana* line DN12 and *D. novamexicana* line N14, and *ebony* and *tan* have been shown to be the causal genes within these QTLs (Lamb et al., 2020; Wittkopp et al., 2009). The Ebony and Tan enzymes catalyze reverse reactions in the melanin/sclerotin pigment biosynthesis pathway (Figure 1b), with Ebony promoting the synthesis of yellow sclerotin pigment and Tan promoting the synthesis of brown and black melanin (Wittkopp & Beldade, 2009).

Pigmentation trends both within and between these two species covary with environmental factors across the United States. The range of the light‐bodied *D. novamexicana* is characterized by higher temperatures, more solar radiation, and less moisture compared to the range of *D. americana* (Davis & Moyle, 2019). Consistent with its desert environment, *D. novamexicana* is significantly more tolerant of desiccation than *D. americana* (Davis & Moyle, 2020). Within *D. americana*, the adaptive cline reported by Wittkopp et al. (2011) showed no association between pigment variation and altitude, mean temperature, or relative humidity, and a manipulative experiment ruled out direct effects of pigmentation on desiccation tolerance. A re‐analysis of that dataset by Clusella‐Trullas and Terblanche (2011), with additional variables, provided support for an association between pigmentation, light, and temperature range: the darker *D. americana* populations, found in the eastern United States, tend to be in locations with lower mean solar radiation and lower diurnal temperature ranges.

The connection between pigment and environmental light is particularly intriguing, because the pigmentation genes *ebony* and *tan* both have pleiotropic effects on fly responses to light (Takahashi, 2013; Figure 1b,c). The Tan enzyme is produced not only in developing cuticles but also in the photoreceptors of the eye, where it processes the inactive compound carcinine (also known as N‐beta‐alanyl histamine) into the neurotransmitter histamine. When a light signal is received, histamine is released by photoreceptors into the synaptic cleft to propagate the signal; from there, it is removed to the associated glial cells, where Ebony converts it back to carcinine, to be returned once more to the photoreceptors (Gavin et al., 2007).

In the model species *D. melanogaster*, both *ebony* and *tan* mutants have abnormal electroretinograms and reduced phototaxis and/or optomotor responses, indicative of impaired vision (Borycz et al., 2002; Chaturvedi et al., 2014; Heisenberg, 1972; Hotta & Benzer, 1969; Pak et al., 1969; Richardt et al., 2002; True et al., 2005). The dark‐colored *ebony* mutants of *D. melanogaster* show reduced mating success relative to wild‐type flies under regular laboratory conditions, but higher mating success than wild‐type flies in dim light (Kyriacou, 1981; Kyriacou et al., 1978; Rendel, 1951), suggesting a possible selective advantage for darker‐colored flies in dim environments.

The same alleles of *ebony* and *tan* that confer lighter, yellower coloration in *D. novamexicana* are also found in some though not all light‐colored populations of *D. americana*, indicating that the genetic basis for light body color is partially shared within and between species (Sramkoski et al., 2020; Wittkopp et al., 2009). This suggested to us that the pleiotropic effects of *ebony* and *tan* on the fly visual system might be similarly shared within and between species. Based on the dual roles of *ebony* and *tan* on fly pigmentation and response to light, and the correlation between high solar radiation and light body color in *D. americana* and *D. novamexicana* (Clusella‐Trullas & Terblanche, 2011; Davis & Moyle, 2019; Table 1), we wondered if behavioral differences in light preference might exist within and between species. We hypothesized that, if differences exist, lighter‐colored flies will tend to prefer more brightly lit environments.

**TABLE 1:** Origins and phenotypes of fly lines used, from darkest to lightest fly line

Species	Line	Full ID	Pigmentation	Collection site	Collection year	Approx. decimal coordinates	Direct Normal Solar Irradiance (kW hr/m^2^/day)
*D. americana*	A04	15010‐0951.04	106.3	Keelers Bay, Lake Champlain, VT	1948	44.7, −73.3	<4.0
*D. americana*	A00	15010‐0951.00	110.8	Anderson, IN	unknown	40.1, −85.7	4.0–4.4
*D. americana*	A01	15010‐0951.01	163.4	Poplar, MT	1947	48.1, −105.2	4.5–4.9
*D. novamexicana*	N14	15010‐1031.14	not measured; visibly lighter than A01	Moab, UT	1949	38.6, −109.6	6.5–6.9

Note

Melanic pigmentation in the *D. americana* lines was measured by Wittkopp et al. (2011) on dissected abdominal cuticles of five male and five female flies, and the least‐squares mean for each line is reported on a scale from 0 (black) to 255 (white). Decimal coordinates are shown as degrees north, degrees west and are estimated from Google Maps. The annual average daily total solar resource for each location was obtained from the National Solar Radiation Database, nsrdb.nrel.gov, using the Direct Normal Solar Irradiance map (https://www.nrel.gov/gis/assets/images/solar‐annual‐dni‐2018‐01.jpg, accessed 17 April 2021).

We tested for light preference across three levels of biological divergence, each of which captures two or more pigment intensity groups:

1. between species;
2. across three different lines of *D. americana*; and
3. between females and males of the same lines.

Based on melanic coloration, we predicted higher light preference in (1) *D. novamexicana* compared to *D. americana*; (2) *D. americana* line A01 compared to lines A00 or A04; and (3) females compared to males.

**FIGURE 2:**
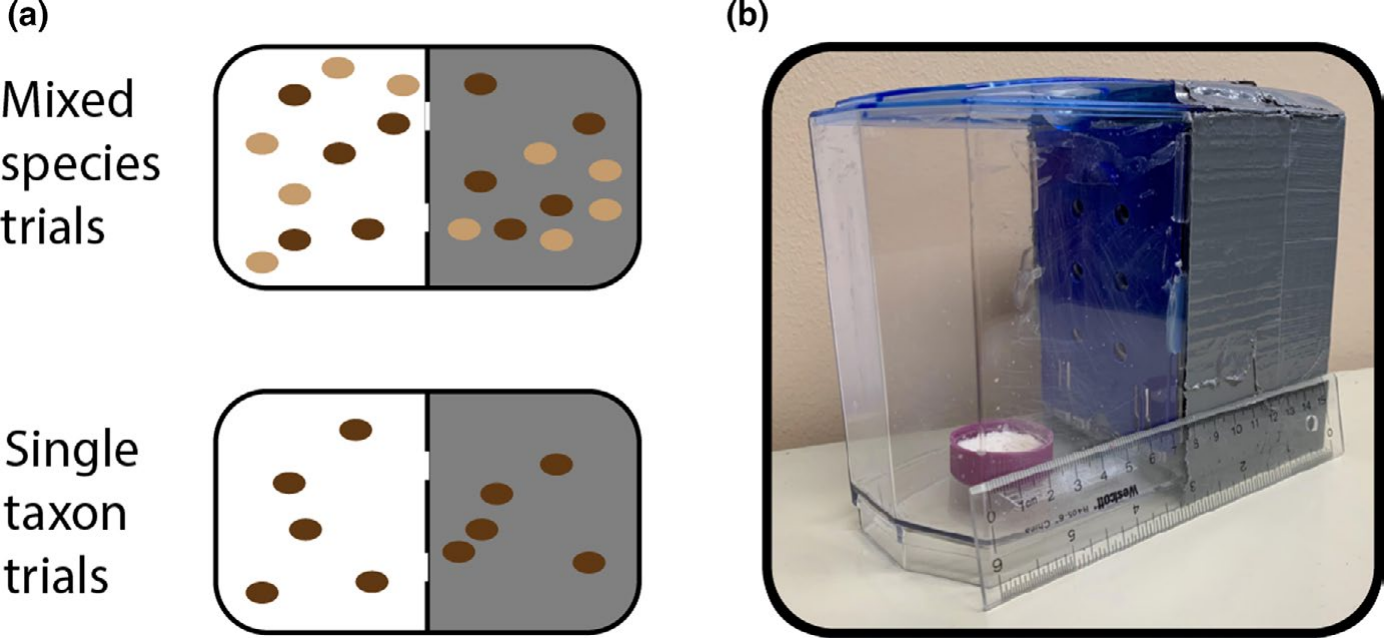
Behavioral choice trials were conducted using “light” versus “dim” artificial habitats. (a) Experimental design for mixed‐species versus single‐taxon experiments. Each cage is divided into a light habitat (white background) and a dim habitat (gray background) and is initially populated with 5 flies of each taxon per side. Dark brown ovals, *D. americana*‐A00. Light brown ovals, *D. novamexicana*‐N14. Drawings not to scale. (b) Fly cage with 15 cm ruler for scale. The purple dish is filled with instant fly food and is matched with a corresponding food dish on the dark side of the cage

In a first round of tests for light preference, male *D. americana* line A00 and male *D. novamexicana* line N14 were placed together into cages containing both light and dark side, with a permeable barrier in between (Figure 2). In a second round of tests, only one type of fly was placed in each cage, and the experiment was expanded to include additional lines and female flies. We counted the number of flies on the light side of each cage over a 6‐day period and tested for effects of taxon and sex on the number of flies in the light habitat. Our data provide preliminary evidence that pigmentation may be correlated with light‐seeking behavior in the *D. americana*–*D. novamexicana* species pair.

## 2 METHODS

### 2.1 Fly lines

*Drosophila americana* lines A04, A00, and A01 and *Drosophila novamexicana* line N14 were ordered from the Cornell University Drosophila Stock Center (Table 1) and maintained at Whitman College on Nutri‐Fly Instant fly food (Genesee Scientific, San Diego, CA, USA). Flies were maintained at ambient light, on benches adjacent to windows.

Within *D. americana*, A01 is the lightest line that has been documented to date, and it contains a *novamexicana*‐like (functionally “light”) allele linked to the *tan* gene, while the dark A00 line contains functionally “dark” alleles at both *ebony* and *tan* (Wittkopp et al., 2009). The dark A04 line is functionally uncharacterized, although it is phenotypically very similar to line A00 (Table 1). *Drosophila novamexicana*‐N14 is the best characterized line of its species (Cooley et al., 2012; Wittkopp et al., 2009), but is actually somewhat dark relative to the range of variation within *D. novamexicana* (see Davis & Moyle, 2019 for images of lighter lines).

### 2.2 Experimental overview

Mixed‐species trials were performed in fall 2017, summer 2018, and spring 2019. For each trial, twenty male flies were placed in each cage: ten on each side, with five *D. americana*‐A00 and five *D. novamexicana*‐N14 on each side (Figure 2a). This number was selected as being easily countable by eye. The number of flies in the “light” habitat was counted at 12 p.m. daily, for 6 days per trial. In 2019, an additional 4 p.m. data collection time was added to assess the effect of time of day on fly behavior.

Single‐taxon trials were performed in the spring, summer, and fall of 2020, across five separate rounds of data collection. For each trial, ten flies were placed in each cage: five on each side, with each cage containing flies from a single line (Figure 2a). The number of flies in the “light” habitat was counted at 12 p.m. daily, for 6 days per trial. Both males and females were tested in the 2020 experiments, but each cage contained only a single sex. Due to the COVID‐19 pandemic, data collection by two of the experimenters was split between work done at Whitman College and work done at the students' homes. In each case, the data were coded as two separate experiments based on their locations.

### 2.3 Cage construction

To provide alternate light environments for the behavioral choice experiments, cages were constructed using small, transparent betta fish tanks with a dark plastic divider (Figure 2b). All outer sides of half of each cage were covered in two layers of duct tape to create a dark environment. Uniform holes ¼” in diameter were drilled into the dividers, allowing flies to pass between the light and dark sides of the cages. The dividers were locked in place by hot glue, sealing them to the insides of the cages. Clear tape was used on the inside of the lids to prevent flies from escaping through airholes. Each side of the container had identical plastic caps filled with synthetic fly food to sustain the flies throughout the trial period. Only enough water was added to the synthetic fly food to slightly saturate it, to prevent the buildup of excess condensation in the cages.

### 2.4 Selection of flies for behavioral trials

To ensure that flies used in the behavioral trials were no more than 1 week old, all adult flies were transferred out of the collecting vials 1 week prior to each trial. On the day of the trial, the collecting vials—containing flies which had eclosed within the past week—were chilled at 4℃ to immobilize the flies. Genital morphology was used to sex the flies, since these species lack both sex combs and sex‐specific pigmentation patterns. Flies of a single sex and taxon were sorted in sets of five into empty test tubes. The vials were kept off ice so liveliness could be evaluated once they warmed up. This was to ensure they had not been damaged and could fly and move normally. Flies that appeared old, deformed, or injured were also returned to the main population. Once collected and checked for liveliness, flies were re‐immobilized by chilling on ice to facilitate transfer and were then poured into each side of the cage. The lids were secured with clear tape.

### 2.5 Data collection in the behavioral trials

In 2017, fly cages were placed in a darkened room under a greenhouse grow light set on a 12‐hr timer. Due to concerns that the artificial light was creating warm temperatures, in all subsequent experiments, fly cages were instead placed on a table about a meter away from a large window, exposing them to natural sunlight.

Each trial was run for six consecutive days. At 12 p.m. every day, the number and species of flies in the light side of each cage were recorded. In the mixed‐species experiments, this was done by looking for the number of dark‐bodied flies (*D. americana*‐A00) and light‐bodied flies (*D. novamexicana*‐N14) present in the light side of the cage. In 2019, a second observation period at 4 p.m. was added.

At the end of each trial, cages were placed in a freezer at −20℃ for 1 hr to immobilize the flies. This allowed us to remove the lid and more thoroughly look for missing or dead flies. The data from cages with dead or missing flies were excluded from analysis. We disposed of the flies and cleaned the cages with ethanol.

### 2.6 Temperature evaluation

In the 2019 experiment, a temperature control study was set up to test for a temperature difference between the light and dark sides of the cages. The wire probes of Fluke 52 II dual input digital thermometers (Everett, WA) were placed in both the light and dark sides of two empty cages. We recorded the temperature reading of each side of each cage, at noon and 4 p.m. daily for 6 days.

### 2.7 Statistical analyses

To test for differences in fly light preference, a generalized linear model was fitted using the glm() command in RStudio 1.3.1093, “Apricot Nasturtium,” within the lme4 package. We assumed a Poisson distribution for the dependent variable, which was the number of flies on the light side of the cage. Independent variables included a fixed effect of taxon; a fixed effect of sex in experiments that included both male and female flies; a fixed effect of time of day for comparisons between 12 p.m. and 4 p.m.; a random effect of cage, to account for the repeated measurements made on each cage; and a random effect of experiment to account for the fact that multiple rounds of data collection were performed, at different times and by different groups of experimenters.

A paired *t* test in R was used to determine whether there was a significant temperature difference between the light and dark sides of the cages.

### 2.8 Genotyping

At the end of the 2020 experiments, the flies were visually inspected to verify homogeneity of pigmentation within each line. To further confirm that the lines had not interbred over the course of the experiments, one female fly of each line was sequenced at both *tan* and *ebony* genes. DNA was extracted using the Omega E.Z.N.A. Tissue DNA Kit (Norcross, GA, USA) and eluted in 50 μl of water. Partial sequence was amplified from the *tan* gene using primers 5′‐ CCGATGCCTGTTCCATTAAC‐3′ and 5′‐ GGCGGCTTGTATTTACCAAA‐3′, and from the *ebony* gene using primers 5′‐AGCCCGAGGTGGACATCA‐3′ and 5′‐GTATGGGTCCCTCGCAGAA‐3′, with G‐Biosciences Taq DNA Polymerase (St. Louis, MO, USA). Thirty cycles of PCR were performed with a 54℃ annealing temperature and a 30‐s extension time. PCR product purity and concentration were estimated from a 1% agarose gel.

Samples were sequenced, using both forward and reverse primers, by Eton Biosciences (San Diego, CA, USA). Manually trimmed sequences were compared to sequences of *D. americana* and *D. novamexicana* obtained from GenBank and from Cooley et al. (2012). Alignments were created in Geneious R9.1 (Biomatters, https://www.geneious.com).

## 3 RESULTS

### 3.1 Mixed‐species male trials show more *D. novamexicana* than *D. americana* in the light habitat

**FIGURE 3:**
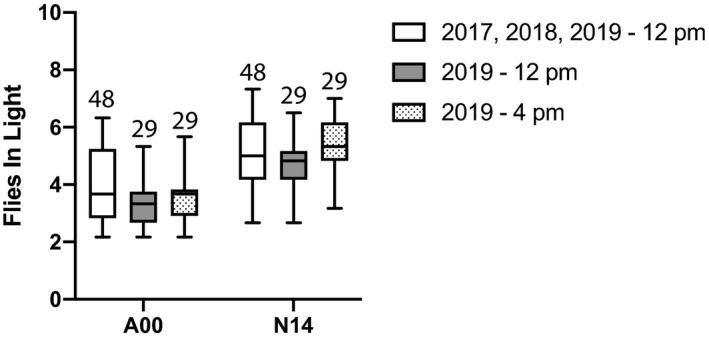
In mixed‐species trials of male flies, *Drosophila americana* line A00 is found less often in the “light” habitat than *D. novamexicana* line N14. The number of successful trials is shown above each data column. A mean value was calculated across the 6 days of each successful trial. Bars represent the range, boxes represent quartiles, and horizontal lines inside the boxes mark the median, for each set of mean values. White bars show the results from 12 p.m. data collection in 2017, 2018, and 2019 combined; *D. novamexicana* was found in the light significantly more often than *D. americana* (*Z* = 6.003; *p* < .001). The gray and dotted bars show only the 2019 data, collected at 12 p.m. and 4 p.m., respectively. Within each collection time, *D. novamexicana* was found in the light significantly more often than *D. americana* (12 p.m.: *Z* = 6.789; *p* < .001; 4 p.m.: *Z* = 8.199; *p* < .001), but there was also a significant effect of data collection time with more flies found in the light habitat at 4 p.m. (*Z* = 2.951; *p* < .01)

In all four mixed‐species datasets (2017, 2018, 2019–12 p.m., and 2019–4 p.m.), more total *D. novamexicana* than *D. americana* were observed on the light sides of the fly cages (Figure 3). The effect of species was significant (Table 2). This result is consistent with our hypothesis that the light‐bodied *D. novamexicana*, which is found in putatively lighter and brighter habitats in the wild, would show a stronger preference for well‐lit environments than the dark‐bodied *D. americana*.

**TABLE 2:** Effects of taxon and sex on fly habitat choice

Experiment(s)	*N*	Source of variation	Estimate	*SE*	*Z*	*p*
2017, 2018, 2019—12 p.m. Males only	48	*D. novamexicana*‐N14	0.23777	0.03961	6.003	<.001
2019, 12 p.m. versus 4 p.m. Males only	29	*D. novamexicana*‐N14 Time of day−4 p.m.	0.39734 0.10841	0.03741 0.03673	10.622 2.951	<.001 <.01
2020—12 p.m. Males and females	372	*D. americana*‐A04 *D. americana*‐A01 *D. novamexicana*‐N14 Sex‐Male	0.06392 0.14115 −0.01975 −0.14367	0.02996 0.03171 0.03081 0.01927	2.134 4.452 −0.641 −7.454	<.05 <.001 ns <.001

Note

Data were collected from each cage once per day for 6 days. Taxon and sex were considered fixed effects; experiment and cage were considered random effects; and the response variable (the number of flies in the “light” habitat each day) was assumed to have a Poisson distribution. A positive *Z*‐value indicates a greater number of flies in the “light” habitat relative to A00 (for effects of taxon); females (for effect of sex); or the 12 p.m. time point (for effect of time of day). *N* = the number of successful 6‐day trials across both sexes and all taxa, with success based on all flies being present and alive at the end of the 6 days. ns, not significant (*p* > .05).

**FIGURE 4:**
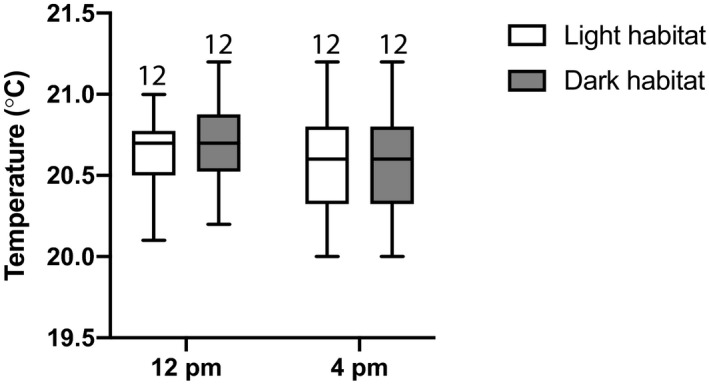
Cage temperature is consistent across habitats. Bars represent the range, boxes represent quartiles, and horizontal lines inside the boxes mark the median. Sample size is shown above each data column. Data were collected once per day, for 6 days, on each of two cages, in 2019. Temperature did not differ significantly between light habitat and dark habitat (paired *t* test: *t* = 0.848, *df* = 23, *p* = .405)

The behavioral difference between species cannot be ascribed to a preference for distinct temperature regimes: the mean difference in temperature between the light and dark habitats was negligible, at both noon and 4 p.m., and not statistically significant (Figure 4; *t* = 0.848, *df* = 3, *p* = .405). Time of day had a significant effect on total numbers of flies in the light habitat (Table 2). Flies of both species were found in the light habitat more often at 4 p.m. than at 12 p.m. (Figure 3). Thus, time of day affected the total numbers of flies on the light side, but did not alter the observed pattern of greater light preference in *D. novamexicana* compared to *D. americana*.

### 3.2 Single‐taxon trials of males and females show varied effects of taxon and a consistent effect of sex

**FIGURE 5:**
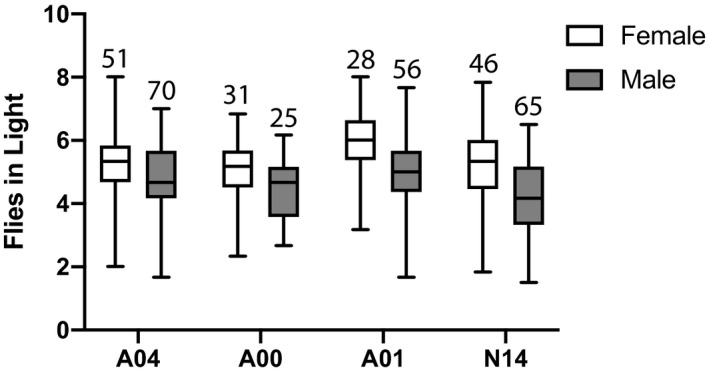
In single‐taxon, single‐sex trials, females are consistently found in the “light” habitat more often than males. Taxa are arranged along the X‐axis from darkest to lightest. Lines A04, A00, and A01 are *D. americana*; line N14 is *D. novamexicana*. The number of successful trials is shown above each data column. Data were collected across five different experiments in 2020, at 12 p.m. daily. A mean value was calculated across the 6 days of each successful trial. Bars represent the range, boxes represent quartiles, and horizontal lines inside the boxes mark the median, for each set of mean values. Males were found less often in the light than females (*Z* = −7.454, *p* < .001). *Drosophila americana*‐A04 and *Drosophila americana*‐A01 were more often in the light habitat than *D. americana*‐A00 (*Z* = 2.134, *p* < .05 and *Z* = 4.452, *p* < .001, respectively), while *D. novamexicana*‐N14 did not differ significantly from *D. americana*‐A00 (*Z* = −0.641, *p* > .05)

In experiments with one taxon per cage, in contrast to the mixed‐species experiments, no significant difference was observed between *D. americana*‐A00 and *D. novamexicana*‐N14 (Table 2). The preference of *D. novamexicana* for the light habitat was similar to that of the dark‐bodied A04 and A00 lines of *D. americana* (Figure 5). Within *D. americana*, the lightest line (A01) was found in the light habitat more often than either of the darker lines (A00, A04).

In the 2020 experiments, a consistent and significant effect of sex was observed (Table 2). Across all four lines of flies utilized, females—which have slightly lighter abdominal pigmentation than males—were observed more often than males in the light habitat (Figure 5).

### 3.3 Fly line genotyping

Sequencing results indicated that all fly lines were homozygous for the expected alleles at both *tan* and *ebony* (Appendices 1 and 2). At the *tan* gene, lines *D. americana*‐A00 and ‐A01 and *D. novamexicana*‐N14 all matched the corresponding sequences found on GenBank. Two SNPs differentiated the *americana* allele from the *novamexicana* allele, in the sequenced region. No GenBank sequence was available for line *D. americana*‐A04, but this sequence contained both of the *americana* SNPs. It also had a unique 12‐bp deletion, in the sixth intron of the gene (Appendix 1). At *ebony*, a short sequence was obtained, containing a SNP that has been shown to differentiate between *D. americana* and *D. novamexicana* (Cooley et al., 2012). The three *americana* lines all had the *americana* allele at this SNP, while *D. novamexicana*‐N14 had the *novamexicana* allele; a second SNP in this region showed the same pattern (Appendix 2).

## 4 DISCUSSION

Correlations between melanin pigmentation and a variety of other phenotypic traits are commonly observed, across vertebrates and insects (San‐Jose & Roulin, 2018). Here, we investigate whether within‐species and between‐species melanic pigmentation differences, in the dark‐bodied *D. americana* and the light‐bodied *Drosophila novamexicana*, are associated with behavioral differences with respect to light. We conducted two sets of experiments. In the first (2017, 2018, and 2019 datasets), male flies of *D. americana* line A00 and *D. novamexicana* line N14 were placed together in behavioral choice cages. These experiments revealed a consistent and highly significant effect of species, with the lighter bodied *D. novamexicana* found slightly but significantly more often in the “light” habitat compared to *D. americana*, for data collected at both mid‐day and afternoon times. In contrast, a second set of experiments with only a single type of fly per cage (the 2020 datasets) did not reveal any difference between *D. americana*‐A00 and *D. novamexicana*‐N14.

These divergent results are not unexpected, given the small overall difference between species combined with variation across experiments. Variation across experiments is expected to occur by chance alone, as well as due to variables such as subtle differences in methodology, and is best addressed through additional replication of the experiment (Nakagawa & Parker, 2015; Nosek & Errington, 2020). *Drosophila novamexicana* line N14 is one of the darker lines of this highly variable species—see Davis and Moyle (2019) for quantification of pigment variability in *D. americana* versus *D. novamexicana* and for an image of the abdominal cuticle from a much lighter *D. novamexicana* individual. Repeating the second set of experiments, using one of the lighter lines of *D. novamexicana*, would create a better opportunity to detect species differences in habitat choice if it is true that light preference and melanic pigmentation are correlated.

Seasonal variation might be expected to influence fly behavior, especially given that seasonality in *Drosophila* appears to depend on a circadian clock (Stoleru et al., 2007) which in turn is influenced by *ebony* (Newby & Jackson, 1991; Suh & Jackson, 2007). While we cannot exclude the effects of seasonality, we note that both of our sets of experiments included fall, spring, and summer data collection efforts.

Alternatively, it is possible that the divergent results are due to the presence versus absence of interspecies interactions. The 2017, 2018, and 2019 datasets included cages populated with both *D. americana* and *D. novamexicana*, while the 2020 datasets featured only one type of fly per cage. Several species of male *Drosophila* have indeed been shown to demonstrate differential patterns of aggression toward conspecific versus heterospecific males (Gupta et al., 2019). However, this finding was primarily observed when the species involved were distantly related, whereas *D. americana* and *D. novamexicana* are sister species thought to have diverged less than 0.5 MYA (Caletka & McAllister, 2004). Additionally, we found that the mixed‐species trials produced a greater species difference in habitat choice compared to single‐taxon trials. In contrast, Gupta et al. (2019) found that aggressive behavior tended to be lower toward heterospecifics than toward conspecifics, which would if anything tend to promote coexistence rather than spatial segregation of the two species.

In a comparison of courtship and mating behaviors in *D. americana* and *D. novamexicana*, Spieth (1951) found that *D. novamexicana* males were more active and aggressive in pursuing mating attempts than *D. americana* males. This could lead to interspecific dynamics impacting the results of the 2017, 2018, and 2019 datasets, although male–male interactions per se were not addressed in that study (Spieth, 1951). Given the relatively small effect of species, and the variation observed across experiments, additional research will be required to determine the robustness and replicability of the species difference documented here.

In our second set of experiments, we explored the effects of intraspecies pigment variation and sex on habitat choice. Pigment variation within *D. americana* was somewhat correlated with habitat choice: the lightest line (A01) was found significantly more often in the light habitat than the two dark lines (A04 and A00). Line A01 has a functionally *D. novamexicana*‐like (“light”) allele at *tan*, but not *ebony*, whereas line A00 has non‐*novamexicana*‐like (“dark”) alleles at both genomic regions (Wittkopp et al., 2009). Given the pleiotropic role of *tan* in recycling histamines in the visual system, it is possible that the A01 “light” allele at the *tan* locus contributes to that line's apparently greater preference for well‐lit habitats. Across *D. americana*, the genetic basis of pigment variation is complex and is only incompletely explained by variation at *tan* and *ebony* (Sramkoski et al., 2020). Future research on the potential pleiotropic effects of *tan* and *ebony* is thus best done on fly lines such as A01 and A00, whose *tan* and *ebony* alleles have been functionally characterized (Wittkopp et al., 2009). Because the genetic basis for pigmentation in the dark line A04 is unknown, and *tan* and *ebony* might not be major contributors, we consider predictions regarding line A04 to be less robust than predictions regarding lines A01 or A00.

Interestingly, our second set of experiments also revealed a significant effect of sex. Female flies were found in the light habitat more often than males, in *D. novamexicana* as well as in all three lines of *D. americana*. Within *D. americana*, females have slightly lighter melanin pigmentation than males (Wittkopp et al., 2011). This finding is, therefore, consistent with our hypothesis that lighter bodied flies will have a correlated preference for lighter habitats. Although many sex‐linked behaviors have been reported in *Drosophila* (Asahina, 2018), sex‐specific differences in light preference have not, to our knowledge, been previously demonstrated.

Overall, our findings in *D. americana* and *D. novamexicana* suggest that correlations may exist between pigmentation and habitat choice between species, within species, and between the sexes, with trends in each case for lighter pigmentation to be associated with a slightly greater preference for a brightly lit environment. Out of seven comparisons made, four support a positive correlation between light body color and light habitat preference; two support a negative correlation; and one supports no correlation (Table 3). The observed correlations, if repeatable, could originate from the pleiotropic nature of the pigmentation and vision genes *tan* and *ebony*, or they could reflect independent evolution of the two traits in response to parallel selective pressures.

**TABLE 3:** Summary of predictions tested

Lighter group	Darker group	Prediction confirmed	Prediction rejected	Inconclusive result
*D. novamexicana*‐N14	*D. americana*‐A00	(a)	–	(b)
*D. novamexicana*‐N14	*D. americana*‐A01	–	(b)	–
*D. americana*‐A01	*D. americana*‐A00	(b)	–	–
*D. americana*‐A01	*D. americana*‐A04	(b)	–	–
*D. novamexicana*‐N14	*D. americana*‐A04	–	(b)	–
Female (x4 lines)	Male (x4 lines)	(b)	–	–

Note

For each comparison, the prediction was considered confirmed if the lighter group was found in the lighter habitat significantly more often than the darker group; rejected if the reverse was true; and inconclusive if no significant difference was observed. a, data from 2017 to 2019 experiments; b, data from 2020 experiments.

A direct test of the pleiotropy hypothesis would be best achieved by transgenic manipulation. If the two traits are correlated due to pleiotropic effects of *tan* and *ebony*, then reducing the function of *tan* should result in lighter bodied flies with greater preference for well‐lit habitats, while reducing the function of *ebony* should have the opposite effects. To assess the hypothesis of parallel selective pressures, in contrast, field experiments will likely be required. Little work has been done on the behavioral ecology of natural *Drosophila* populations (but see Soto‐Yéber et al., 2018), and the light and color environments directly experienced by *D. americana* and *D. novamexicana* in the wild have not yet been quantified.

The work presented here is one of few behavioral studies of these two species (but see Spieth, 1951) and the first demonstration to our knowledge of a sex‐specific difference in preference for environmental light in *Drosophila*. Given the variation of our findings for *D. novamexicana* between our two experimental designs, additional replication will be necessary to evaluate the correlations that we observed between pigmentation and behavior. However, the majority of our comparisons suggest a pattern in which lighter bodied flies tend to exhibit preference for a more brightly lit environment. Two genes, *tan* and *ebony*, together explain most of the color difference between the dark‐bodied *D. americana*‐DN12 and the lighter bodied *D. novamexicana*‐N14 (Lamb et al., 2020; Wittkopp et al., 2009) and are also required for visual function (Heisenberg, 1972; Takahashi, 2013; True et al., 2005). We propose that the pleiotropic nature of *tan* and *ebony* may have shaped evolutionary change in both pigmentation and light preference—potentially within as well as between these two closely related yet intriguingly divergent species.

## CONFLICT OF INTEREST

None declared.

## AUTHOR CONTRIBUTIONS

**Arielle M. Cooley:** Formal analysis (lead); funding acquisition (lead); methodology (equal); project administration (lead); supervision (lead); visualization (lead); writing–review and editing (lead). **Suzanne Schmitz:** Investigation (lead); writing–original draft (supporting). **Eduardo J. Cabrera:** Investigation (equal); writing–original draft (supporting). **Mitchell Cutter:** Conceptualization (equal); methodology (equal). **Maxwell Sheffield:** Conceptualization (equal); methodology (equal). **Ian Gingerich:** Investigation (equal); writing–original draft (supporting). **Gabriella Thomas:** Investigation (equal); writing–original draft (supporting). **Calvin N. M. Lincoln:** Investigation (equal); writing–original draft (supporting). **Virginia H. Moore:** Investigation (equal); writing–original draft (supporting). **Alexandra E. Moore:** Investigation (equal); writing–original draft (supporting). **Sarah A. Davidson:** Investigation (equal); writing–original draft (supporting). **Nikhil Lonberg:** Investigation (equal); writing–original draft (supporting). **Eli B. Fournier:** Investigation (equal); writing–original draft (supporting). **Sophia M. Love:** Investigation (lead); writing–original draft (supporting). **Galen Posch:** Investigation (equal); writing–original draft (supporting). **Matthew B. Bihrle:** Investigation (equal); writing–original draft (supporting). **Spencer D. Mayer:** Investigation (equal); writing–original draft (supporting). **Kuenzang Om:** Conceptualization (equal); methodology (equal). **Lauren Wilson:** Investigation (equal); writing–original draft (supporting). **Casey Q. Doe:** Investigation (equal); writing–original draft (supporting). **Chantalle E. Vincent:** Investigation (lead); writing–original draft (supporting). **Elizabeth R. T. Wong:** Investigation (equal); writing–original draft (supporting). **Ilona Wall:** Investigation (equal). **Jarred Wicks:** Investigation (lead); writing–original draft (supporting). **Stephon Roberts:** Investigation (lead); writing–original draft (supporting).

### OPEN RESEARCH BADGES

This article has earned an Open Data Badge for making publicly available the digitally‐shareable data necessary to reproduce the reported results. The data are available at https://doi.org/10.5061/dryad.dv41ns1xz.

## Data Availability

DNA sequences greater than 200 bp in length are available on GenBank (BankIt submission 2483022, sequence IDs MZ577312‐MZ577319), and all DNA sequences obtained in this experiment are shown in Appendices 1 and 2.

## APPENDIX 1

Alignment of partial sequences from the *tan* gene. The sequence without a chromatogram was obtained from GenBank; the rest were obtained by PCR and direct sequencing as described in the Methods. Green arrows, PCR primers. Grey arrows, exons. Red boxes enclose *novamexicana* alleles at divergent sites and light blue boxes enclose *americana* alleles at the same sites. FWD, sequence obtained using the forward primer as the sequencing primer. REV, sequence obtained using the reverse primer as the sequencing primer. Line N14 is *D. novamexicana*; lines A01, A00, and A04 are *D. americana*.

## APPENDIX 2

Alignment of partial sequences from an exon of the *ebony* gene. The sequences without chromatograms were obtained from GenBank; the rest were obtained by PCR and direct sequencing as described in the Methods. Green arrows, PCR primers. Red boxes enclose *novamexicana* alleles at divergent sites and light blue boxes enclose *americana* alleles at the same sites. FWD, sequence obtained using the forward primer as the sequencing primer. REV, sequence obtained using the reverse primer as the sequencing primer. Line N14 is *D. novamexicana*; lines A01, A00, and A04 are *D. americana*.

## References

[ece37998-bib-0045] Armbruster, W. A., & Schwaegerle, K. E. (1996). Causes of covariation of phenotypic traits among populations. Journal of Evolutionary Biology, 9(3), 261–276.

[ece37998-bib-0001] Asahina, K. (2018). Sex differences in *Drosophila* behavior: Qualitative and quantitative dimorphism. Current Opinion in Physiology, 6, 35–45. 30386833 10.1016/j.cophys.2018.04.004 PMC6205217

[ece37998-bib-0002] Borycz, J., Borycz, J. A., Loubani, M., & Meinertzhagen, A. N. D. I. A. (2002). *Tan* and *ebony* genes regulate a novel pathway for transmitter metabolism at fly photoreceptor terminals. Journal of Neuroscience, 22, 10549–10557. 12486147 10.1523/JNEUROSCI.22-24-10549.2002 PMC6758454

[ece37998-bib-0003] Caletka, B. C., & McAllister, A. N. D. B. F. (2004). A genealogical view of chromosomal evolution and species delimitation in the *Drosophila virilis* species subgroup. Molecular Phylogenetics and Evolution, 33, 664–670. 10.1016/j.ympev.2004.08.007 15522794

[ece37998-bib-0004] Chaturvedi, R., Reddig, K., & Li, A. N. D. H. S. (2014). Long‐distance mechanism of neurotransmitter recycling mediated by glial network facilitates visual function in *Drosophila* . Proceedings of the National Academy of Sciences, 111(7), 2812–2817. 10.1073/pnas.1323714111 PMC3932938 24550312

[ece37998-bib-0005] Clusella Trullas, S., van Wyk, J. H., & Spotila, A. N. D. J. R. (2007). Thermal melanism in ectotherms. Journal of Thermal Biology, 32, 235–245. 10.1016/j.jtherbio.2007.01.013

[ece37998-bib-0006] Clusella‐Trullas, S., & Terblanche, J. S. (2011). Local adaptation for body color in Drosophila americana: Commentary on Wittkopp et al Heredity, 106, 904–905. 10.1038/hdy.2010.141 21063435 PMC3186221

[ece37998-bib-0007] Cooley, A. M., Shefner, L., Mclaughlin, W. N., Stewart, E. E., & Wittkopp, A. N. D. P. W. (2012). *The ontogeny of color: Developmenta*l origins of pigment divergence in Drosophila americana and D. novamexicana. Evolution & Development, 14, 317–325. 22765203 10.1111/j.1525-142X.2012.00550.x PMC3402224

[ece37998-bib-0008] Davis, J. S., & Moyle, A. N. D. L. C. (2019). Desiccation resistance and pigmentation variation reflects bioclimatic differences in the *Drosophila americana* species complex. BMC Evolutionary Biology, 19, 1–14. 10.1186/s12862-019-1536-7 31694548 PMC6836511

[ece37998-bib-0009] Davis, J. S., & Moyle, A. N. D. L. C. (2020). Constitutive and plastic gene expression variation associated with desiccation resistance differences in the *Drosophila americana* species group. Genes, 11, 146. 10.3390/genes11020146 PMC7073762 32019054

[ece37998-bib-0010] Endler, J. A. (1986). Natural selection in the wild. Princeton University Press.

[ece37998-bib-0011] Gavin, B. A., Arruda, S. E., & Dolph, A. N. D. P. J. (2007). The role of carcinine in signaling at the *Drosophila* photoreceptor synapse. PLoS Genetics, 3, e206. 10.1371/journal.pgen.0030206 18069895 PMC2134947

[ece37998-bib-0012] Gompel, N., & Carroll, A. N. D. S. B. (2003). Genetic mechanisms and constraints governing the evolution of correlated traits in drosophilid flies. Nature, 424, 931–935. 10.1038/nature01787 12931186

[ece37998-bib-0013] Gupta, T., Howe, S. E., Zorman, M. L., & Lockwood, A. N. D. B. L. (2019). Aggression and discrimination among closely versus distantly related species of *Drosophila* . Royal Society Open Science, 6, 190069. 31312482 10.1098/rsos.190069 PMC6599796

[ece37998-bib-0014] Heisenberg, M. (1972). Comparative behavioral studies on two visual mutants of *Drosophila* . Journal of Comparative Physiology, 80, 119–136. 10.1007/BF00696485

[ece37998-bib-0015] Hotta, Y., & Benzer, A. N. D. S. (1969). Abnormal electroretinograms in visual mutants of *Drosophila* . Nature, 222, 354–356. 10.1038/222354a0 5782111

[ece37998-bib-0016] Kyriacou, C. P. (1981). The relationship between locomotor activity and sexual behaviour in *ebony* strains of *Drosophila melanogaster* . Animal Behavior, 29, 462–471. 10.1016/S0003-3472(81)80106-6

[ece37998-bib-0017] Kyriacou, C. P., Burnet, B., & Connolly, A. N. D. K. (1978). The behavioural basis of overdominance in competitive mating success at the *ebony* locus of *Drosophila melanogaster* . Animal Behavior, 26, 1195–1206. 10.1016/0003-3472(78)90109-4

[ece37998-bib-0018] Lamb, A. M., Wang, Z., Simmer, P., Chung, H., & Wittkopp, A. N. D. P. J. (2020). *ebony* affects pigmentation divergence and cuticular hydrocarbons in *Drosophila americana* and *D. novamexicana* . Frontiers in Ecology and Evolution, 8. 10.3389/fevo.2020.00184 PMC10077920 37035752

[ece37998-bib-0019] Lande, R., & Arnold, S. J. (1983). The measurement of selection on correlated characters. Evolution, 37, 1210–1226. 10.1111/j.1558-5646.1983.tb00236.x 28556011

[ece37998-bib-0020] Morales‐Hojas, R., Reis, M., Vieira, C. P., & Vieira, A. N. D. J. (2011). Resolving the phylogenetic relationships and evolutionary history of the *Drosophila virilis* group using multilocus data. Molecular Phylogenetics and Evolution, 60, 249–258. 10.1016/j.ympev.2011.04.022 21571080

[ece37998-bib-0021] Nakagawa, S., & Parker, A. N. D. T. H. (2015). Replicating research in ecology and evolution: Feasibility, incentives, and the cost‐benefit conundrum. BMC Biology, 13, 88. 26510635 10.1186/s12915-015-0196-3 PMC4624660

[ece37998-bib-0022] Newby, L. M., & Jackson, A. N. D. F. R. (1991). *Drosophila ebony* mutants have altered circadian activity rhythms but normal eclosion rhythms. Journal of Neurogenetics, 7, 85–101. 1903161 10.3109/01677069109066213

[ece37998-bib-0023] Nosek, B. A., & Errington, A. N. D. T. M. (2020). What is replication? PLOS Biology, 18, e3000691. 10.1371/journal.pbio.3000691 32218571 PMC7100931

[ece37998-bib-0024] Pak, W. L., Grossfield, J., & White, A. N. D. N. V. (1969). Nonphototactic mutants in a study of vision of *Drosophila* . Nature, 222, 351–354. 10.1038/222351a0 5782110

[ece37998-bib-0025] Pool, J. E., & Aquadro, A. N. D. C. F. (2007). The genetic basis of adaptive pigmentation variation in *Drosophila melanogaster* . Molecular Ecology, 16, 2844–2851. 10.1111/j.1365-294X.2007.03324.x 17614900 PMC2650379

[ece37998-bib-0026] Rajpurohit, S., & Nedved, A. N. D. O. (2013). Clinal variation in fitness related traits in tropical drosophilids of the Indian subcontinent. Journal of Thermal Biology, 38, 345–354. 10.1016/j.jtherbio.2013.04.004

[ece37998-bib-0027] Rajpurohit, S., Parkash, R., & Ramniwas, A. N. D. S. (2008). Body melanization and its adaptive role in thermoregulation and tolerance against desiccating conditions in drosophilids. Entomological Research, 38, 49–60. 10.1111/j.1748-5967.2008.00129.x

[ece37998-bib-0028] Rendel, J. M. (1951). Mating of *ebony, vestigial,* and wild type *Drosophila melanogaster* in light and dark. Evolution, 5, 226–230.

[ece37998-bib-0029] Richardt, A., Rybak, J., Störtkuhl, K. F., Meinertzhagen, I. A., & Hovemann, A. N. D. B. T. (2002). Ebony protein in the *Drosophila* nervous system: Optic neuropile expression in glial cells. Journal of Comparative Neurology, 452, 93–102. 10.1002/cne.10360 12205712

[ece37998-bib-0030] San‐Jose, L. M., & Roulin, A. N. D. A. (2018). Toward understanding the repeated occurrence of associations between melanin‐based coloration and multiple phenotypes. The American Naturalist, 192, 111–130. 10.1086/698010 30016163

[ece37998-bib-0031] Soto‐Yéber, L., Soto‐Ortiz, J., Godoy, P., & Godoy‐Herrera, A. N. D. R. (2018). The behavior of adult *Drosophila* in the wild. PLoS One, 13, e0209917. 10.1371/journal.pone.0209917 30596767 PMC6312304

[ece37998-bib-0032] Spieth, H. T. (1951). Mating behavior and sexual isolation in the *Drosophila virilis* species group. Behaviour, 3, 105–144. 10.1163/156853951X00232

[ece37998-bib-0033] Sramkoski, L. L., McLaughlin, W. N., Cooley, A. M., Yuan, D. C., John, A., & Wittkopp, A. N. D. P. J. (2020). Genetic architecture of a body colour cline in *Drosophila americana* . Molecular Ecology, 29, 2840–2854. 32603541 10.1111/mec.15531 PMC7482988

[ece37998-bib-0034] Stearns, S. C. (1992). The evolution of life histories. Oxford University Press.

[ece37998-bib-0035] Stoleru, D., Nawathean, P., Fernández, M. D. L. P., Menet, J. S., Ceriani, M. F., & Rosbash, M. (2007). The *Drosophila* circadian network is a seasonal timer. Cell, 129, 207–219. 10.1016/j.cell.2007.02.038 17418796

[ece37998-bib-0036] Suh, J., & Jackson, A. N. D. F. R. (2007). *Drosophila ebony* activity is required in glia for the circadian regulation of locomotor activity. Neuron, 55, 435–447. 10.1016/j.neuron.2007.06.038 17678856 PMC2034310

[ece37998-bib-0037] Takahashi, A. (2013). Pigmentation and behavior: Potential association through pleiotropic genes in *Drosophila* . Genes & Genetic Systems, 88, 165–174. 24025245 10.1266/ggs.88.165

[ece37998-bib-0038] Telonis‐Scott, M., Hoffmann, A. A., & Sgrò, A. N. D. C. M. (2011). *The molecular genetics of cl*inal variation: A case study of ebony and thoracic trident pigmentation in Drosophila melanogaster from eastern Australia. Molecular Ecology, 20, 2100–2110. 10.1111/j.1365-294X.2011.05089.x 21466604

[ece37998-bib-0039] Throckmorton, L. H. (1982). The *virilis* species group. In M. Ashburner, H. L. Carson, & A. N. D. J. N. Thompson (Eds.), The genetics and biology of *Drosophila* (Vol. 3b). Academic Press.

[ece37998-bib-0040] True, J. R. (2003). Insect melanism: The molecules matter. Trends in Ecology & Evolution, 18, 640–647. 10.1016/j.tree.2003.09.006

[ece37998-bib-0041] True, J. R., Yeh, S.‐D., Hovemann, B. T., Kemme, T., Meinertzhagen, I. A., Edwards, T. N., Liou, S.‐R., Han, Q., & Li, J. (2005). *Dro*sophila tan encodes a novel hydrolase required in pigmentation and vision. PLoS Genetics, 1, 551–562. 10.1371/journal.pgen.0010063 PMC1285064 16299587

[ece37998-bib-0042] Wittkopp, P. J., & Beldade, A. N. D. P. (2009). Development and evolution of insect pigmentation: Genetic mechanisms and the potential consequences of pleiotropy. Seminars in Cell & Developmental Biology, 20, 65–71. 10.1016/j.semcdb.2008.10.002 18977308

[ece37998-bib-0043] Wittkopp, P. J., Smith‐Winberry, G., Arnold, L. L., Thompson, E. M., Cooley, A. M., Yuan, D., Song, Q., & McAllister, A. N. D. B. F. (2011). *Local adaptation for* body color in Drosophila americana. Heredity, 106, 592–602. 10.1038/hdy.2010.90 20606690 PMC3183901

[ece37998-bib-0044] Wittkopp, P. J., Stewart, E. E., Arnold, L. L., Neidert, A. H., Haerum, B. K., Thompson, E. M., Akhras, S., Smith‐Winberry, G., & Shefner, L. (2009). Intraspecific polymorphism to interspecific divergence: Genetics of pigmentation in Drosophila. Science, 326, 540–544. 10.1126/science.1176980 19900891

